# Comparison of capture-based mtDNA sequencing performance between MGI and illumina sequencing platforms in various sample types

**DOI:** 10.1186/s12864-023-09938-6

**Published:** 2024-01-08

**Authors:** Zehui Feng, Fan Peng, Fanfan Xie, Yang Liu, Huanqin Zhang, Jing Ma, Jinliang Xing, Xu Guo

**Affiliations:** 1 State Key Laboratory of Holistic Integrative Management of Gastrointestinal Cancers and, Department of Physiology and Pathophysiology, Fourth Military Medical University, Xi’an, 710032 China; 2 Department of Obstetrics and Gynecology, Xijing Hospital, Fourth Military Medical University, Xi’an, 710032 China; 3 Department of Clinical Diagnosis, Tangdu Hospital, Fourth Military Medical University, Xi’an, 710038 China; 4 Yanbian University Medical College, Yanji, 133002 China

**Keywords:** DNBSEQ-T7, NovaSeq 6000, Sequencing platform, Capture-Based mtDNA Sequencing

## Abstract

**Background:**

Mitochondrial genome abnormalities can lead to mitochondrial dysfunction, which in turn affects cellular biology and is closely associated with the development of various diseases. The demand for mitochondrial DNA (mtDNA) sequencing has been increasing, and Illumina and MGI are two commonly used sequencing platforms for capture-based mtDNA sequencing. However, there is currently no systematic comparison of mtDNA sequencing performance between these two platforms. To address this gap, we compared the performance of capture-based mtDNA sequencing between Illumina's NovaSeq 6000 and MGI's DNBSEQ-T7 using tissue, peripheral blood mononuclear cell (PBMC), formalin-fixed paraffin-embedded (FFPE) tissue, plasma, and urine samples.

**Results:**

Our analysis indicated a high degree of consistency between the two platforms in terms of sequencing quality, GC content, and coverage. In terms of data output, DNBSEQ-T7 showed higher rates of clean data and duplication compared to NovaSeq 6000. Conversely, the amount of mtDNA data obtained by per gigabyte sequencing data was significantly lower in DNBSEQ-T7 compared to NovaSeq 6000. In terms of detection mtDNA copy number, both platforms exhibited good consistency in all sample types. When it comes to detection of mtDNA mutations in tissue, FFPE, and PBMC samples, the two platforms also showed good consistency. However, when detecting mtDNA mutations in plasma and urine samples, significant differenceof themutation number detected was observed between the two platforms. For mtDNA sequencing of plasma and urine samples, a wider range of DNA fragment size distribution was found in NovaSeq 6000 when compared to DNBSEQ-T7. Additionally, two platforms exhibited different characteristics of mtDNA fragment end preference.

**Conclusions:**

In summary, the two platforms generally showed good consistency in capture-based mtDNA sequencing. However, it is necessary to consider the data preferences generated by two sequencing platforms when plasma and urine samples were analyzed.

**Supplementary Information:**

The online version contains supplementary material available at 10.1186/s12864-023-09938-6.

## Background

Due to rapid technological advancements, second-generation sequencing platforms now have the capability to generate large volumes of short-read data at a comparatively low cost [1]. Illumina's Next-Generation Sequencing (NGS) machines have long maintained their dominance in the sequencing market owing to their high accuracy and throughput [2]. In recent years, MGI Tech, Inc. has introduced several DNBSEQ platforms that incorporate innovative technologies such as DNA nanosphere and the combined probe anchored polymerization (cPAS) sequencing [3]. DNBSEQ demonstrates superior cost-effectiveness compared to the Illumina platform, considering their similar sequencing throughput. Additionally, both platforms exhibit comparable read lengths [4]. The emergence of MGI's DNBSEQ platforms offers researchers an alternative choice when selecting a sequencing platform. Although existing studies have primarily focused on comparing Illumina's platforms with those of MGI in whole genome sequencing, transcriptomics sequencing, and microbiome sequencing, there is currently no study available that specifically compares these two platforms for mitochondrial genome sequencing [5–7].

Mitochondria, the double membrane-bound organelles present in mammalian cells, play essential roles in cell metabolism and contribute to various cellular processes such as apoptosis and calcium signaling [8]. Mitochondrial genomic abnormalities can lead to disruption in mitochondrial functions [9]. Amounting reports link mitochondrial mutations or copy number variations to the occurrence and progression of various diseases [10–12]. The short length and high copy number of mtDNA in plasma make it a potential biomarker in the field of liquid biopsy for tumors [13]. Consequently, there is an increasing demand for mtDNA sequencing. In our previous studies, we have established a capture-based mtDNA sequencing method based on the Illumina platform. With the emergence of the MGI platform, it is possible to achieve mtDNA sequencing data using a similar library preparation process. However, there have been no reports comparing the performance of capture-based mtDNA sequencing between these two sequencing platforms.

Therefore, in the present study, we systematically compared the performance of capture-based mitochondrial DNA sequencing between Illumina's NovaSeq 6000 and MGI's DNBSEQ-T7 platform for various sample types. Our study provides useful recommendations for selection of mtDNA sequencing platforms in different scenarios.

## Material and methods

### Sample collection and DNA extraction

In this study, a total of 50 samples were collected from 36 patients diagnosed with ovarian cancer or hepatocellular carcinoma. These samples included 13 fresh tissue samples, 13 unpaired FFPE tissue samples, 12 PBMC samples, and 6 paired fresh and FFPE tumor tissues. Furthermore, plasma samples were obtained from 10 patients diagnosed with hepatocellular carcinoma, while urine samples were obtained from 10 patients with bladder cancer. In addition, the tumor tissue from 3 patients with hepatocellular carcinoma were also collected for whole transcriptome sequencing. Detailed subject information was provided in Table S1. The ENZA DNA Kit (Omega) and QIAamp DNA FFPE Kit (Qiagen) was used for genomic DNA extraction of the isolated peripheral blood mononuclear cells and fresh tissue samples and FFPE samples, respectively. The QIAamp Circulating Nucleic Acid Kit (Qiagen) was used forthe extraction of cell-free DNA (cfDNA). All DNA samples were quantified using the Qubit 4.0 fluorometer (Thermo Fisher).

### Library construction

Prior to library construction, genomic DNA extracted from fresh tumor tissue, FFPE samples, and PBMCs was fragmented using a focused ultrasonicator (Scientz98, Ningbo, China). Subsequently, DNA fragments ranging from 300 to 500 bp in size were selected, end-repaired, ligated with sequencing adapters, amplified, and captured using biotinylated mtDNA probes. The VAHTSTM Universal DNA Library Prep Kit for Illumina® V3 and for MGI was employed as the library building reagent for the NovaSeq 6000 and the DNBSEQ-T7 platforms, respectively. The purified beads utilized in both platforms were identical and referred to as Novizan VAHTSTM DNA Clean Beads (Vazyme #N411).

### Capture-based mtDNA sequencing

Our study employed a hybridization capture-based approach due to its superior depth coverage uniformity compared to a PCR-based method [14]. Specifically, we conducted capture-based mtDNA sequencing using custom-designed biotinylated probes as previously reported [15, 16]. In brief, the prepared whole-genome sequencing (WGS) libraries were hybridized with the homemade biotinylated capture probes. The reaction system was supplemented with binding buffer containing streptavidin-coated magnetic beads. For the DNBSEQ-T7 platform, the captured mtDNA libraries undergo additional circularization. Subsequently, the captured mtDNA libraries were amplified and subjected to 150 bp paired-end sequencing on the NovaSeq 6000 (Illumina) and the DNBSEQ-T7 platforms, respectively.

### RNA extraction, library construction and sequencing

Fresh tissue samples were used to extract total RNA using the AP-MN-MS-RNA-250 kit (axygen). The QIAseq FastSelect rRNA HMR Kit (Qiagen) was applied to remove rRNA prior to library construction. Subsequently, the QIAseq Stranded Total RNA Kit (Qiagen) was utilized for library preparation. The libraries were then sequenced using the NovaSeq 6000 and the DNBSEQ-T7 platforms, respectively.

### Characteristic analysis of mtDNA sequencing data

The raw data underwent trimming using fastp (v0.20.1) [17] to remove low-quality reads and adaptor contamination. FastQC (v0.12.1) [18] was used to evaluate the quality and GC content of the mtDNA reads. Clean reads were aligned to the rCRS and hg19 references using bwa-mem (v0.7.17) [19], and duplicated reads were removed using Picard MarkDuplicates (v1.81). GATK IndelRealigner (v3.2–2) [20] was applied for indel realignment. To eliminate contamination from NUMTs, we only retained the read pairs, which is properly and uniquely mapped to mitochondrial genome, to detect the mtDNA mutations. SAMtools (v1.7) [21] was used to generate pileup files for mtDNA mutation calling. To accurately call mtDNA mutations, we applied several filtering conditions based on established criteria [14]. These included: 1) requiring a minimum of three reads supporting the alternative allele in each strand, 2) ensuring a total site sequencing coverage of at least 100X, 3) setting a variant allele fraction (VAF) threshold of ≥ 2% on both strands, and 4) removing heterogeneity sites in rCRS repeat regions (66–71, 303–316, 513–525, 5892, 3106–3107, 12418–12425, 16182–16194). We also excluded variants with C:G > A:T transversions (VAF ≤ 10%) to avoid potential artifacts related to 8-oxoguanine [22]. In addition to these filtering measures, the heteroplasmy level of each mtDNA mutation was calculated as the number of variant reads divided by the number of total reads. We specifically detected mtDNA mutations with a VAF ≥ 2% in our study. To ensure consistent read depth for the same sample across both platforms, we utilized the Picard DownSampleSam tool. The relative coverage of each position was calculated using SAMtools (v1.7) and normalized by the total depth of the whole mitochondrial genome multiplied by 1 Mio (Million). In addition, we used MitoTool, a tool based on phylogenetic methods, to determine the mtDNA haplotype (haplogroup) of each sample [23, 24]. The haplotypes (haplogroups) of all samples were shown in table S2.

The following formula was used to estimate mtDNA copy number [16],

$$\text{CN} = \frac{\text{ mtDNA average sequencing depth}}{\text{average sequencing}\text{ depth of reference gene}}\text{X 2}$$

Here, CN represents the mtDNA copy number. The mtDNA average sequencing depth refers to the mean coverage depths of the entire mtDNA genome. The average sequencing depth of the reference gene indicates the mean coverage depths of six specific locations in the nuclear genome (nDNA).

The fragment lengths of mtDNA were determined using the Picard CollectInsertSizeMetrics tool. For each mtDNA fragment, the first nucleotide at the 5’ end was recorded. The proportions of A-end, T-end, G-end, and C-end fragments were calculated using the following formula: specific-end fragments divided by the sum of all A-end, G-end, C-end, and T-end fragments. Additionally, the 5’ end motifs were normalized based on the base composition of the mitochondria reference genome [25].

### mtDNA analysis based on transcriptomic data

The raw RNA-seq data were trimmed using fastp (v0.20.1) [17] with minimum length set to 15nt. After trimming, clean reads were aligned to the hg38 human genome using HISAT2 [26] with ‘–rna-strandness RF’ parameters. SAMtools (v1.7) were used to extract the reads, which were properly mapped to the mitochondrial genome. The relative coverage per base of each strand (light-strand and heavy-strand) was calculated using SAMtools (v1.7) and normalized by the total depth of whole mitochondrial genome multiplied by 1 Mio.

### Statistic analysis

Statistical analyses and depiction of the graphs were conducted using GraphPad Prism version 8.3.0. The statistical tests employed in this study included the Wilcoxon rank-sum test and paired t-test. Pearson correlation analysis was used to assess the correlations of the depth distribution between the two platforms. All statistical analyses were two-sided, and *P*-values less than 0.05 were considered statistically significant.

## Results

### Comparison of data quality control parameters between DNBSEQ-T7 and NovaSeq 6000 platforms

**Fig. 1:**
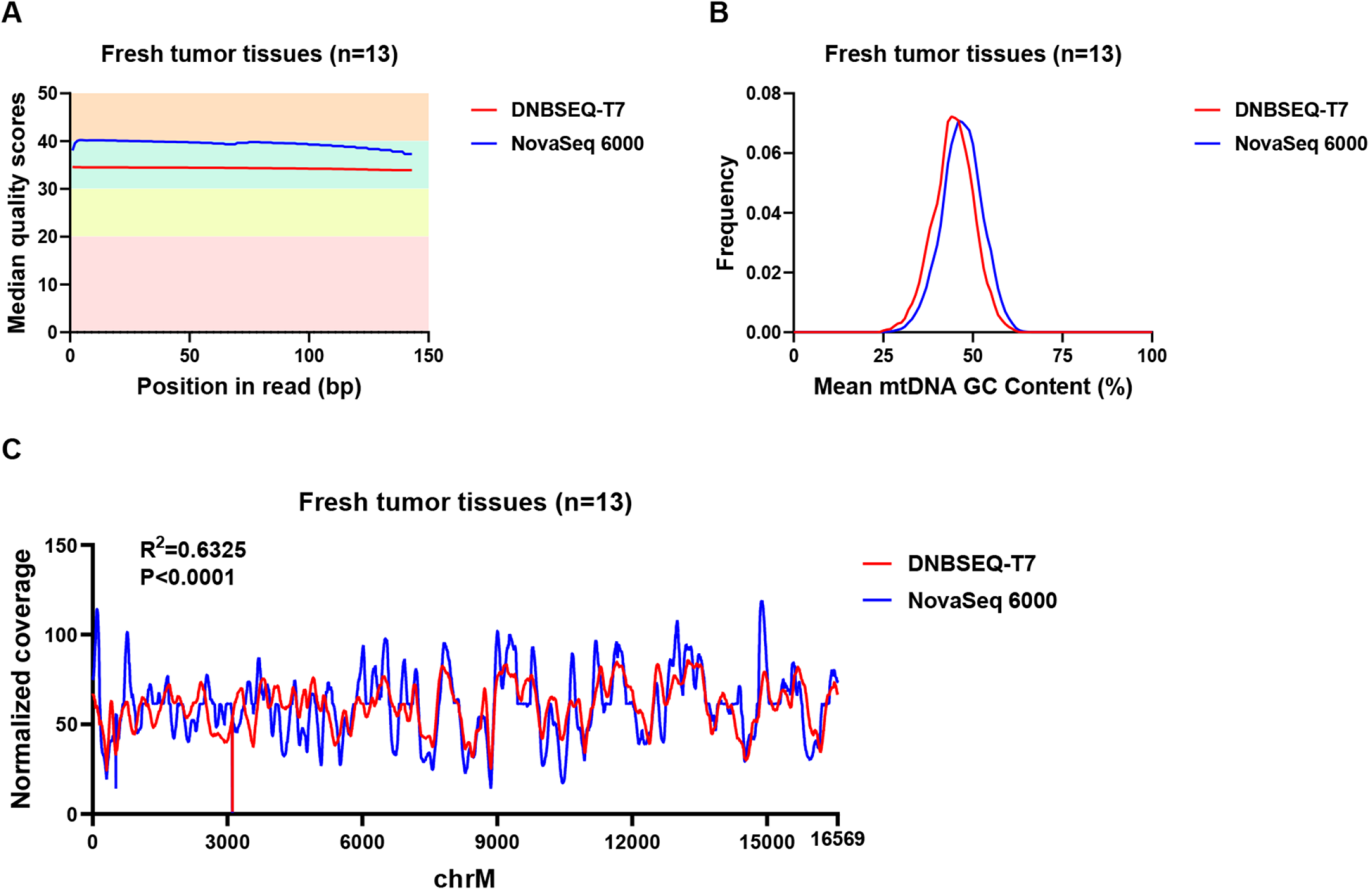
Data quality control parameters for fresh tumor tissues between DNBSEQ-T7 and NovaSeq 6000 platforms. **A**. Comparison of the base sequencing quality values between two platforms. **B**. Comparison of the mtDNA GC content between the two platforms. **C**. Comparison of the sequencing the depth distribution between the two platforms

To evaluate the quality of mtDNA data obtained from the NovaSeq 6000 and DNBSEQ-T7 platforms, we utilized the FastQC software for fastp file quality checking. In terms of sequencing quality in fresh tumor tissue (as depicted in Fig. 1A), we defined low-quality reads as those with a sequencing quality score < 30. No obvious strand bias was observed between two platforms or among five sample types. Both platforms exhibited a similar result, with quality scores surpassing Q30. As shown in Figure S1, this pattern was consistently observed across other sample types. Moreover, as depicted in Fig. 1B, the mtDNA GC content in fresh tumor tissue closely mirrored the GC content of the mtDNA reference sequence (approximately 44%) on both platforms. This similarity was consistently observed across various sample types, as illustrated in Figure S2. In various sample types, NovaSeq 6000 exhibited slightly higher mtDNA GC content compared to DNBSEQ-T7. To determine the sequencing depth at each locus, we employed Samtools to calculate the depth and subsequently analyzed the depth of each site after normalization. High correlation coefficients between both platforms were observed in all sample types (Fig. 1C and Figure S3).

### Comparison of mtDNA data output ratio between DNBSEQ-T7 and NovaSeq 6000 platforms

**Fig. 2:**
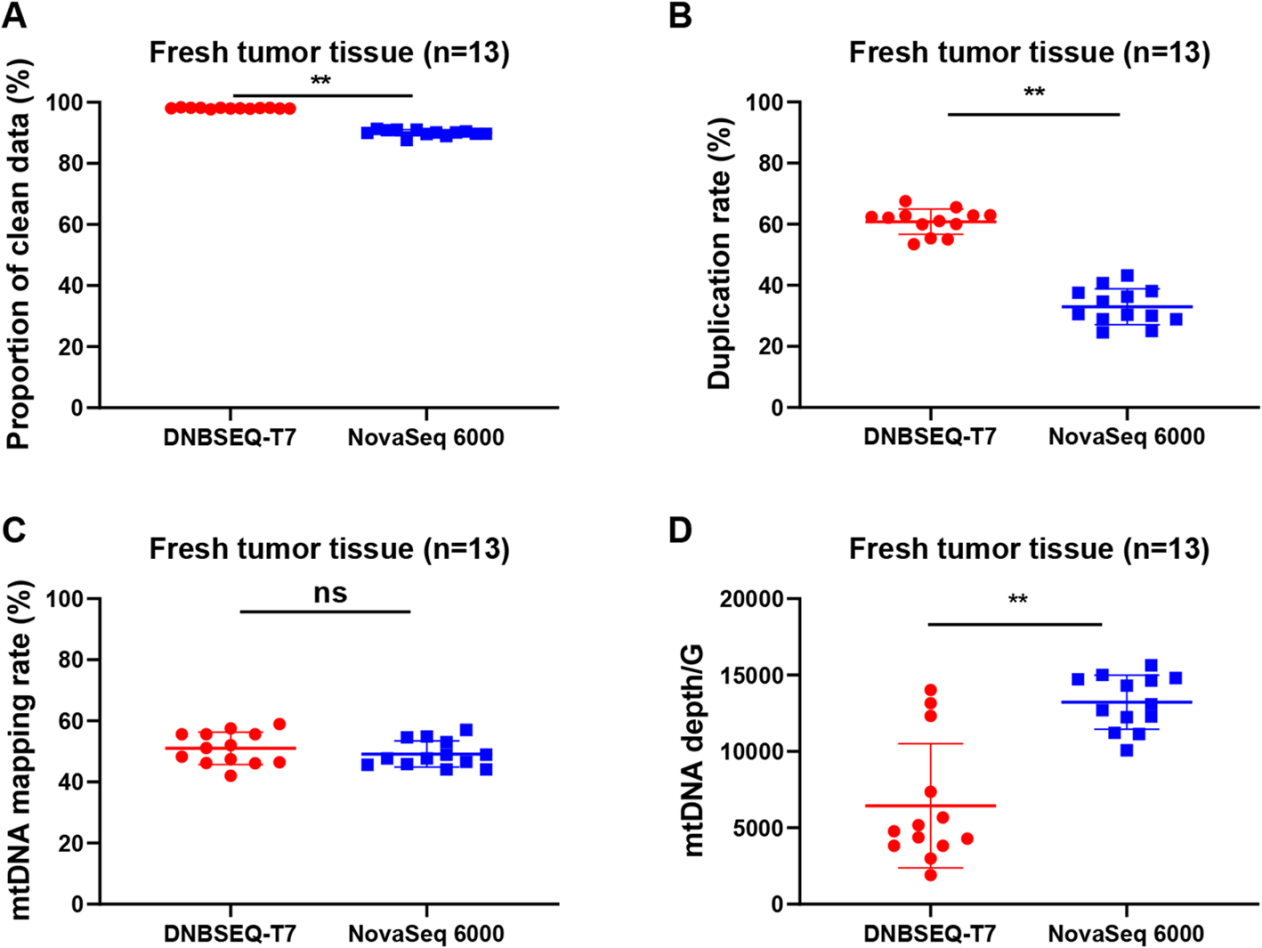
mtDNA data output ratio for fresh tumor tissues between the two platforms. **A**-**D**. Comparison of the proportion of clean data, the duplication rate, the mtDNA mapping rate, the normalized amount of sequencing data and the corresponding sequencing depth in free tumor tissues between the two platforms

The effective utilization of raw data for both platforms was calculated. As depicted in Fig. 2A, the proportion of clean data was higher in DNBSEQ-T7 compared to NovaSeq 6000, specifically for fresh tumor tissue samples. This characteristic remained consistent in other sample types (Figure S4). To assess the duplication rate in the two sequencing platforms, we focused on identifying exact duplicates, which are identical sequence copies derived from raw sequence data. As illustrated in Fig. 2B, DNBSEQ-T7 exhibited a higher duplicate ratio compared to NovaSeq 6000. The consistent results were also observed in other sample types (Figure S5). Capture efficiency between the two platforms was further analyzed (Fig. 2C). Our data demonstrated that there were no statistically significant differences in mapping rates between the two platforms. Similar observation held true for other sample types (Figure S6). Moreover, the evaluation of the normalized amount of sequencing data and the corresponding sequencing depth was carried out. As presented in Fig. 2D, DNBSEQ-T7 exhibited a lower sequencing depth per Gigabyte data compared to NovaSeq 6000 in fresh tissue samples. Similar results were observed in other sample types (Figure S7).

### Comparison of mtDNA copy number in two platforms

**Fig. 3:**
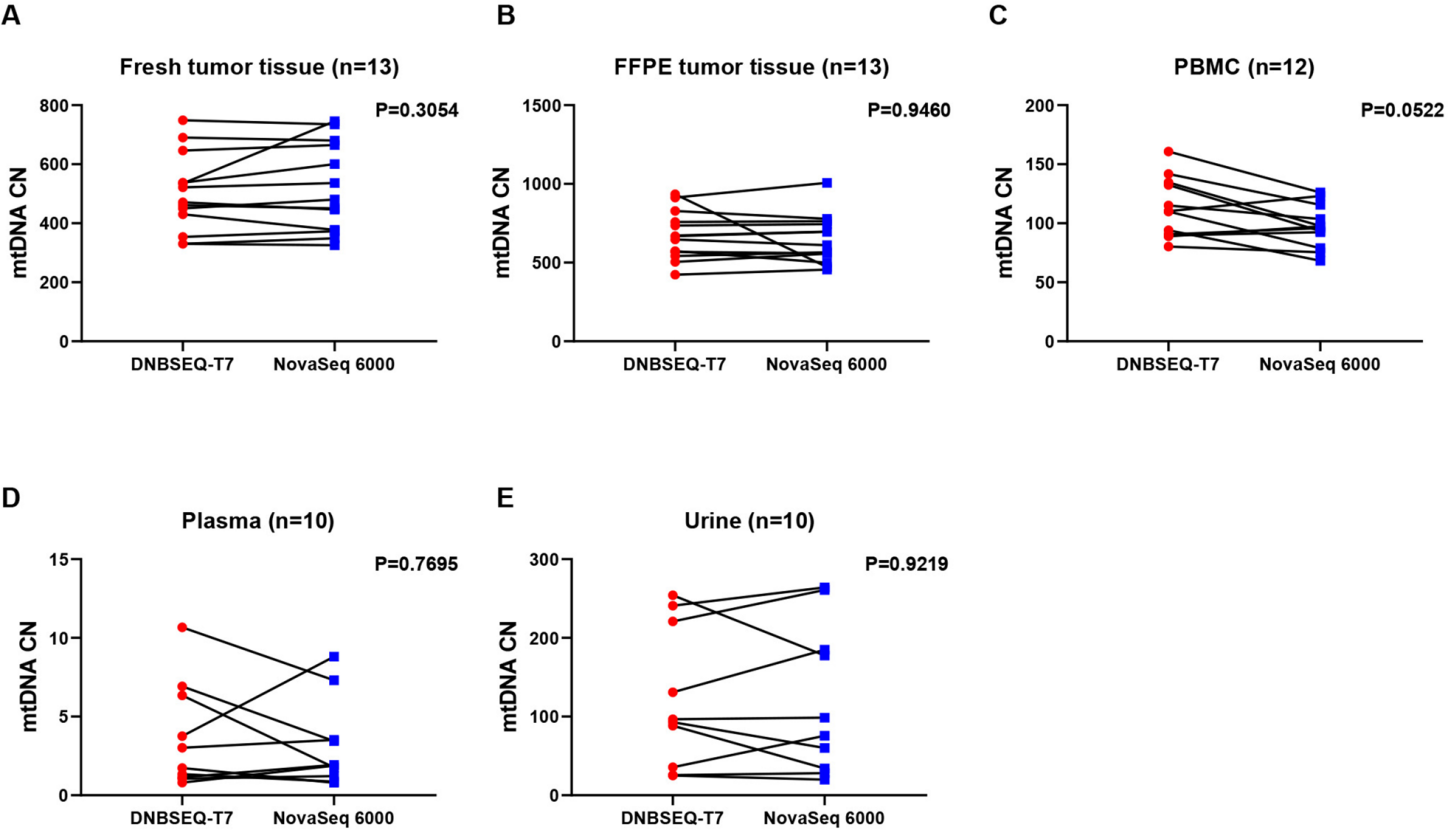
Comparison of mtDNA copy number among five different sample types between two platforms. **A-E**. mtDNA copy number in fresh tumor tissues, formalin-fixed paraffin-embedded (FFPE) tumor tissues, Peripheral blood mononuclear cells (PBMCs), plasma and urine samples

Numerous studies have highlighted the significant role of the altered mtDNA copy number in various common disorders. Therefore, we compared the mtDNA copy numbers of several sample types between the two platforms. As depicted in Fig. 3, the two platforms exhibited good consistency of the mtDNA copy number among fresh tissue, FFPE tissue, plasma, and urine samples.

### Comparative analysis of mtDNA mutation profiles between the two platforms

**Fig. 4:**
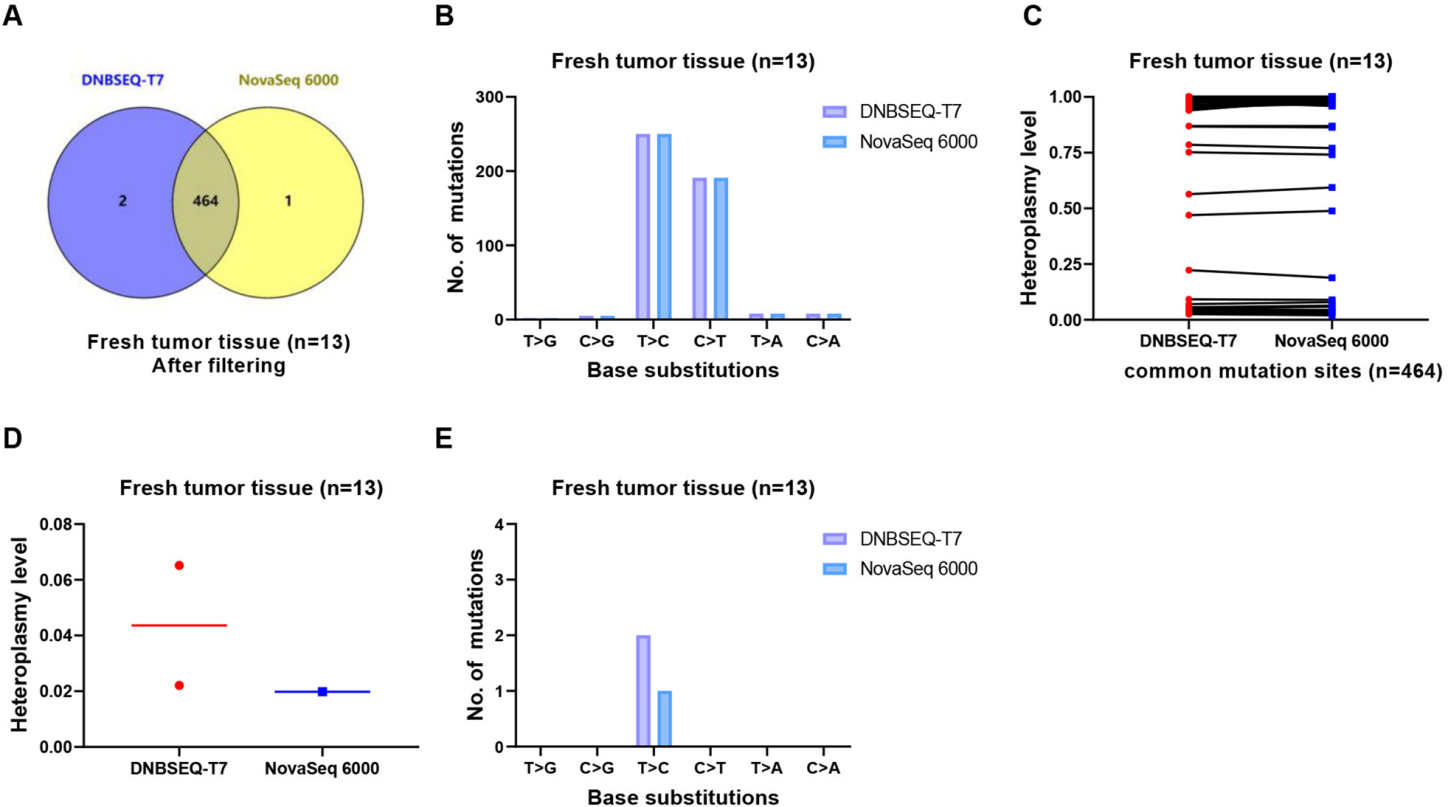
Comparison of mtDNA mutation profiles for fresh tumor tissues between the two platforms. **A**-**C**. Comparison of the mutation number, the base substitution and the heteroplasmy level between the two platforms. **D-E**. Comparison of the mutation density and the base substitution of platform-derived mutations between the two platforms

To achieve consistency, we extracted the same depth data for both platforms based on the sample with the lowest depth, ensuring that each sample had the same sequencing depth across platforms. The minimum allele frequency (MAF) on both strands was set at ≥ 2% during mutation calling. As shown in Fig. 4A, the majority of mtDNA mutations in fresh tissue samples were detectable on both platforms, although each platform generated a small number of platform-derived mutations. Therefore, a high consistency was observed in the observed base substitution patterns of mtDNA mutations (Fig. 4B). Additionally, the level of heterogeneity in these mutations was also highly comparable between the two platforms (Fig. 4C). In fresh tissue, FFPE tissue, and PBMC samples, the occurrence of platform-derived mutations was relatively low (Fig. 4D-E, Figure S8 and S9). However, both platforms detected a significant number of platform-derived mutations in plasma and urine samples (Figure S10 and S11). A complete list of mtDNA variants has been provided in the revised Table S3. In addition, we included 6 cases of paired fresh and FFPE tumor tissues, all subjected to sequencing on the Illumina platform. Notably, we observed a high degree of concordance when comparing homogeneous/heterogeneous variants between fresh and FFPE tumor tissues. The results for these paired samples have been included in Table S4.

### The fragment size distribution of cf-mtDNA in plasma and urine samples between the two platforms

**Fig. 5:**
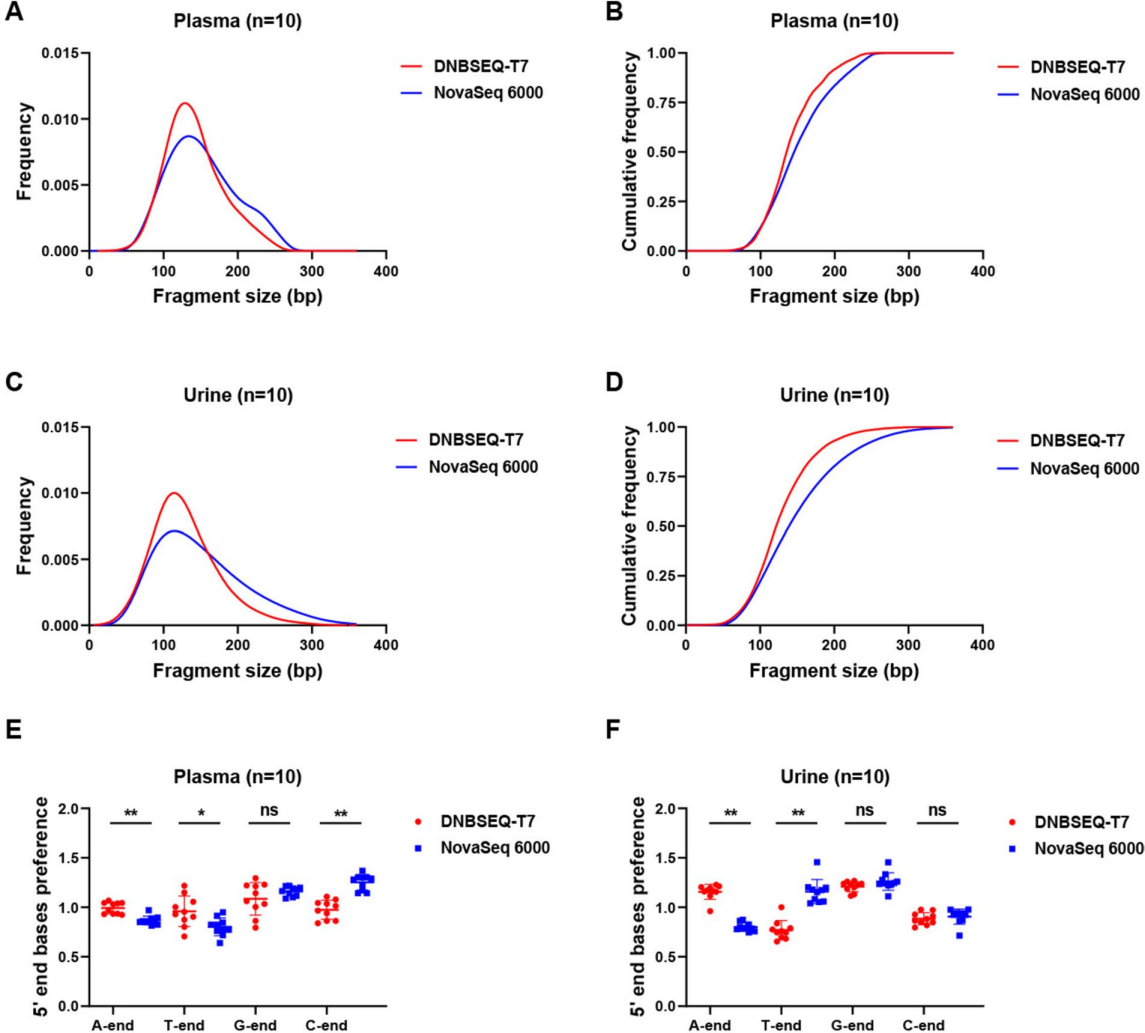
Comparison of cf-mtDNA characteristics in plasma and urine samples between the two platforms. **A-D**. Comparison of distributions and cumulative frequency plots of fragment size in plasma and urine cf-mtDNA between two platforms. **E–F**. Comparison of the 5’ ends base preference in plasma and urine cf-mtDNA between two platforms

We next examined whether there were variances in the size distribution of cf-mtDNA fragments in plasma and urine samples sequencedon the two platforms. Intriguingly, as illustrated in Fig. 5, we observed that NovaSeq 6000 exhibited greater detectable range of fragment sizes than DNBSEQ-T7. Furthermore, proportion of > 158 bp fragment detected by NovaSeq 6000 was notably higher than that by DNBSEQ-T7. To further characterize the fragmentation pattern, we evaluated the preferred ends at the 5' ends of cf-mtDNA fragments. As depicted in Fig. 5E-F, the proportion of base components at the cf-mtDNA ends was similar between the two platforms in plasma samples. However, in urine samples, statistically significant differences were observed in the content of A-ends, T-ends, and C-ends. Specifically, Novaseq 6000 exhibited a relatively higher proportion of T-ends, while DNBSEQ-T7 displayed a relatively higher proportion of A-ends.

### Comparison of the depth distribution in transcriptome data between the two platforms

**Fig. 6:**
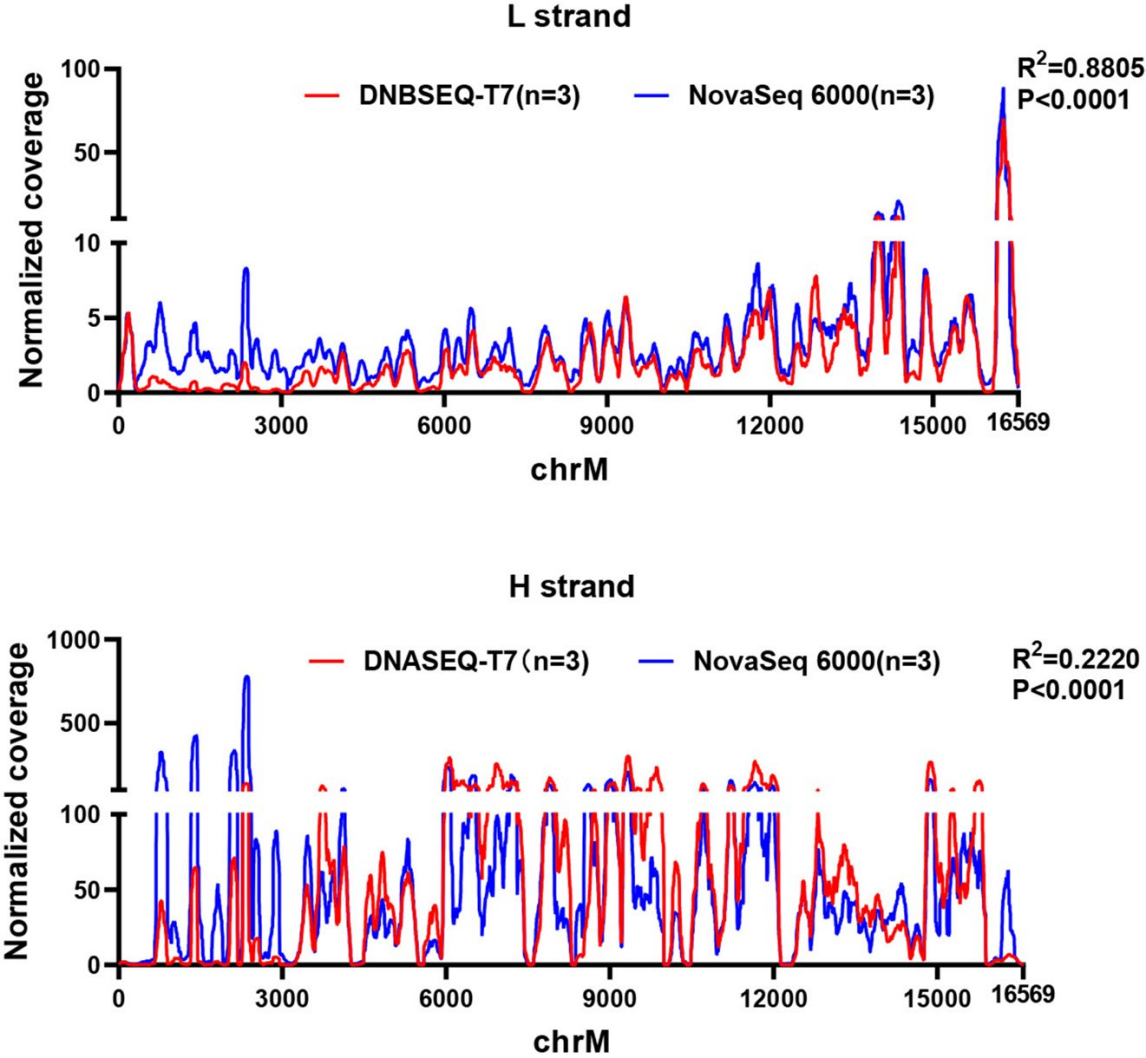
A consistent depth distribution of mtDNA derived from transcriptome data between the two platforms. **A**. Comparison of the mtDNA depth distribution in the light chains between two platforms. **B**. Comparison of the mtDNA depth distribution in the heavy chains between two platforms

In addition to capture-based mtDNA sequencing, we extracted mtDNA reads from the transcriptome data and compared the performance of the two platforms in obtaining mtDNA data. The analysis of the mtDNA depth distribution was carried out in the transcriptome data, specifically focusing on the light and heavy chains where the depth was normalized across 16,569 sites. As illustrated in Fig. 6, a high correlation coefficient was observed between the mtDNA depth distributions of the two platforms, with a significant Pearson correlation level of *P* < 0.001. These findings indicate the consistent depth distribution of mtDNA derived from transcriptome data between the two platforms.

## Discussion

In recent years, with the advance of sequencing technologies, the cost of sequencing has been decreasing and the demand for mtDNA sequencing in medicine has been increasing [27]. Therefore, utilizing next-generation sequencing technologies for mtDNA sequencing has become a routine practice. Among the most commonly used platforms in the market are Illumina's NovaSeq 6000 and MGI's DNBSEQ-T7. Comparing the performance of these two platforms in capture-based mtDNA sequencing is of great importance for clinical applications involving mtDNA detection. In this study, capture-based mtDNA sequencingwere conducted in different types of samples with the NovaSeq 6000 and DNBSEQ-T7 platforms. First, the sequencing quality of all types of samples was compared between the two platforms. Our data indicate that both platforms provide high-quality sequencing data regardless of the sample type. Furthermore, the mtDNA GC content of both platforms is close to the mtDNA reference (GC content is 44%), indicating no significant bias.

Additionally, the comparative analysis of the cost-effective performance was conducted between the two platforms. The DNBSEQ-T7 platform shows a higher proportion of clean data compared to the NovaSeq 6000, while it also exhibits a higher duplication rate, which aligns with previous findings [2]. However, after normalizing the amount of sequencing data, we found that the NovaSeq 6000 exhibited the higher mtDNA depth per gigabase (Gb) data than DNBSEQ-T7. Consequently, although MGI's current sequencing cost is lower, it generates less clean mtDNA data. To acquire an equivalent amount of clean data, the overall cost seems to be similar between the two platforms. Furthermore, the introduction of new platforms such as Illumina's NovaSeq X Plus is expected to further drive down costs.

Numerous studies have emphasized the significant role of the altered mitochondrial DNA (mtDNA) copy number and mutations in various common disorders, including cancer [28, 29]. Our study consistently observed comparable performance between platforms when comparing mtDNA copy numbers across different sample types. However, it is crucial to consider the sample type when mutation was detected. We clearly observed platform-derived low-frequency mutations in plasma and urine samples, with the majority of their frequencies around 2%. These mutations can be attributed to factors such as insufficient sequencing depth, platform-derived biases, and differences in algorithmic approaches. Further comparative analyses can provide valuable insights into the specific causes and offer guidance for improving mutation detection methods.

In recent years, rapid advance has been reported in liquid biopsy, particularly the use of cfDNA fragmentation patterns in tumor detection [30–32]. However, there are certain differences in characteristics of plasma and urine cf-mtDNA fragment detected on the two platforms. In our study, the fragment size distribution and preferred ends was examined, indicating that the NovaSeq 6000 platform had a wider detectable range for cf-mtDNA fragments compared to the DNBSEQ-T7 platform [5]. The proportion of C-ends and G-ends in plasma cf-mtDNA is significantly higher than that of A-ends and T-ends in NovaSeq 6000, which is consistent with the previous observations [33]. However, the differences in the proportions of the four nucleotide ends in plasma cf-mtDNA are not significant in DNBSEQ-T7. A significant difference was found in the proportions of A-ends and T-ends of urine cf-mtDNA between the two platforms. Therefore, both the fragment size distribution and end preference of cf-mtDNA can be influenced by the differences in sequencing platform chemistry, library preparation methods, data processing, and analysis algorithms. Consequently, when cfDNA fragment characteristics was utilized for clinical practices, it is crucial to consider the impact of different sequencing platforms.

To further assess the performance of these two platforms in mtDNA sequencing, we extracted mtDNA data from transcriptome and compared the coverage of the mitochondrial genome's heavy (H) strand and light (L) strand. The results showed a high degree of similarity between the two platforms.

In summary, the performance of the NovaSeq 6000 and DNBSEQ-T7 platforms is comparable for capture-based mtDNA sequencing. However, the choice of sequencing platform for mtDNA sequencing in specific cases still requires comprehensive consideration. Firstly, for sequencing cost, sequencing platforms are constantly evolving, leading to a continuous reduction in sequencing costs. For instance, the introduction of Illumina's Novaseq X and X Plus has further lowered the existing costs. Secondly, both platforms generate a significant number of inconsistent low-frequency mutations when analyzing plasma and urine samples, which may impact mutation analysis in liquid biopsy applications. Finally, there are variations between the two platforms in fragmentomic analysis, necessitating careful consideration when selecting the appropriate sequencing platform for such analyses.

### Supplementary Information

**Additional file 1: Table S1.** Sample and sequencing information. **Table S2.** A list of consensus haplotypes from all samples. **Table S3.** A complete list of mtDNA variants. **Table S4.** Homoplasmic/heteroplasmic fraction of mtDNA variants in fresh and FFPE tumor tissue.

**Additional file 2: Figure S1.** Comparison of the base sequencing quality values among four sample types between the two platforms.** Figure S2.** Comparison of the mtDNA GC content among four different sample types between the two platforms.** Figure S3. **Comparison of the depth distribution among four different sample types between the two platforms.** Figure S4. **Comparison of the proportion of clean data among four sample types between the two platforms.** Figure S5. **Comparison of the duplication rate among four different sample types between the two platforms.** Figure S6. **Comparison of the mtDNA mapping rate among four different sample types between the two platforms.** Figure S7. **Comparison of the normalized amount of sequencing data and the corresponding sequencing depth.** Figure S8. **Comparison of mtDNA mutation profiles for FFPE samples.** Figure S9. **Comparison of mtDNA mutation profiles for PBMC samples.** Figure S10. **Comparison of mtDNA mutation profiles for plasma samples.** Figure S11. **Comparison of mtDNA mutation profiles for urine samples.

## Data Availability

The sequencing data have been uploaded to the Genome Sequence Archive for Human (GSA-Human) under accession PRJCA019746 (http://bigd.big.ac.cn/gsa-human.). The following is a link for editors and reviewers to view, http://bigd.big.ac.cn/gvm/getProjectFile?t=69f8ad67.
