# Supplementary material for: Comparison of capture-based mtDNA sequencing performance between MGI and illumina sequencing platforms in various sample types
Source: BMC Genomics. 2024 Jan 8;25:41. doi: 10.1186/s12864-023-09938-6 (PMC10773069; doi:10.1186/s12864-023-09938-6)
Supplement: Supplementary file 2 — Additional file 2: Figure S1. Comparison of the base sequencing quality values among four sample types between the two platforms. Figure S2. Comparison of the mtDNA GC content among four different sample types between the two platforms. Figure S3. Comparison of the depth distribution among four different sample types between the two platforms. Figure S4. Comparison of the proportion of clean data among four sample types between the two platforms. Figure S5. Comparison of the duplication rate among four different sample types between the two platforms. Figure S6. Comparison of the mtDNA mapping rate among four different sample types between the two platforms. Figure S7. Comparison of the normalized amount of sequencing data and the corresponding sequencing depth. Figure S8. Comparison of mtDNA mutation profiles for FFPE samples. Figure S9. Comparison of mtDNA mutation profiles for PBMC samples. Figure S10. Comparison of mtDNA mutation profiles for plasma samples. Figure S11. Comparison of mtDNA mutation profiles for urine samples. [file 12864_2023_9938_MOESM2_ESM.docx]

**Figure S1. Comparison of the base sequencing quality values among four sample types between the two platforms**

**
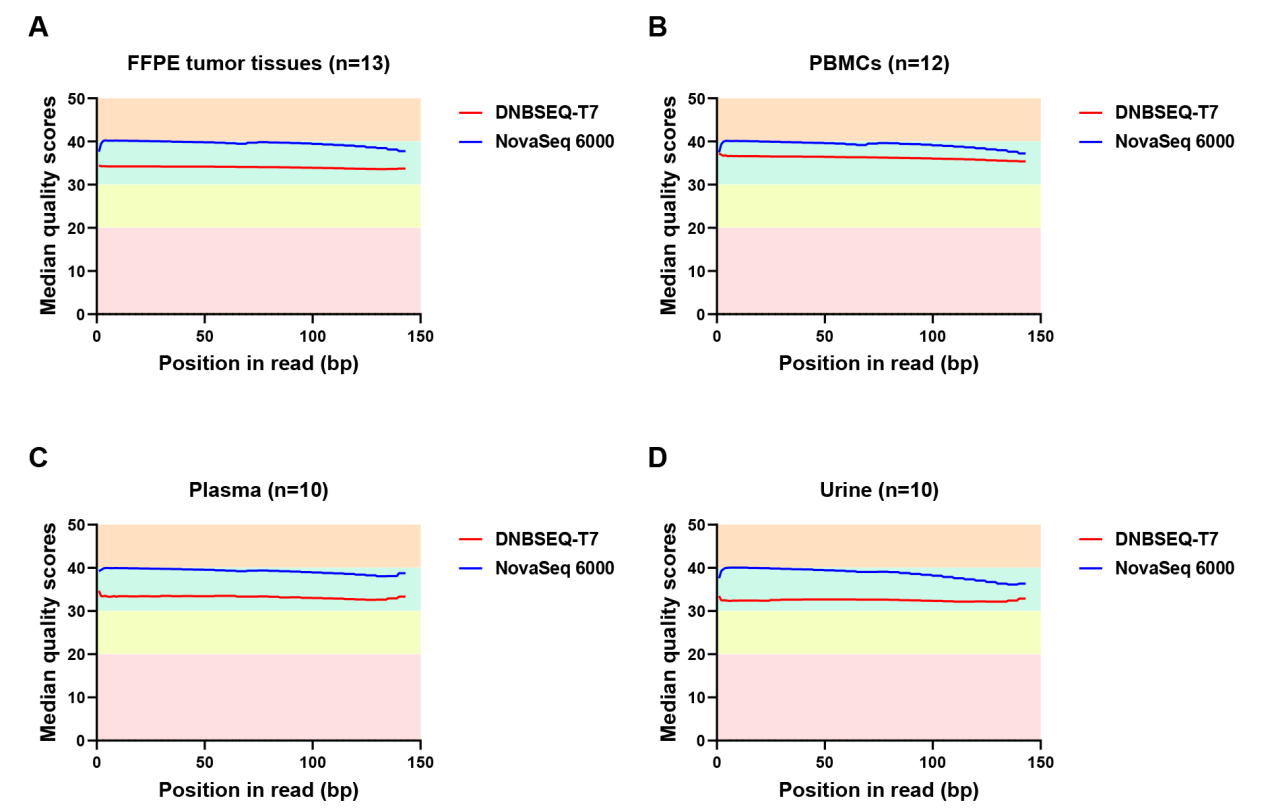
**

A-D. Comparison of the base sequencing quality values for formalin-fixed paraffin-embedded (FFPE), peripheral blood mononuclear cells (PBMCs), plasma and urine samples between the two platforms.

**Figure S2. Comparison of the mtDNA GC content among four different sample types between the two platforms**

**
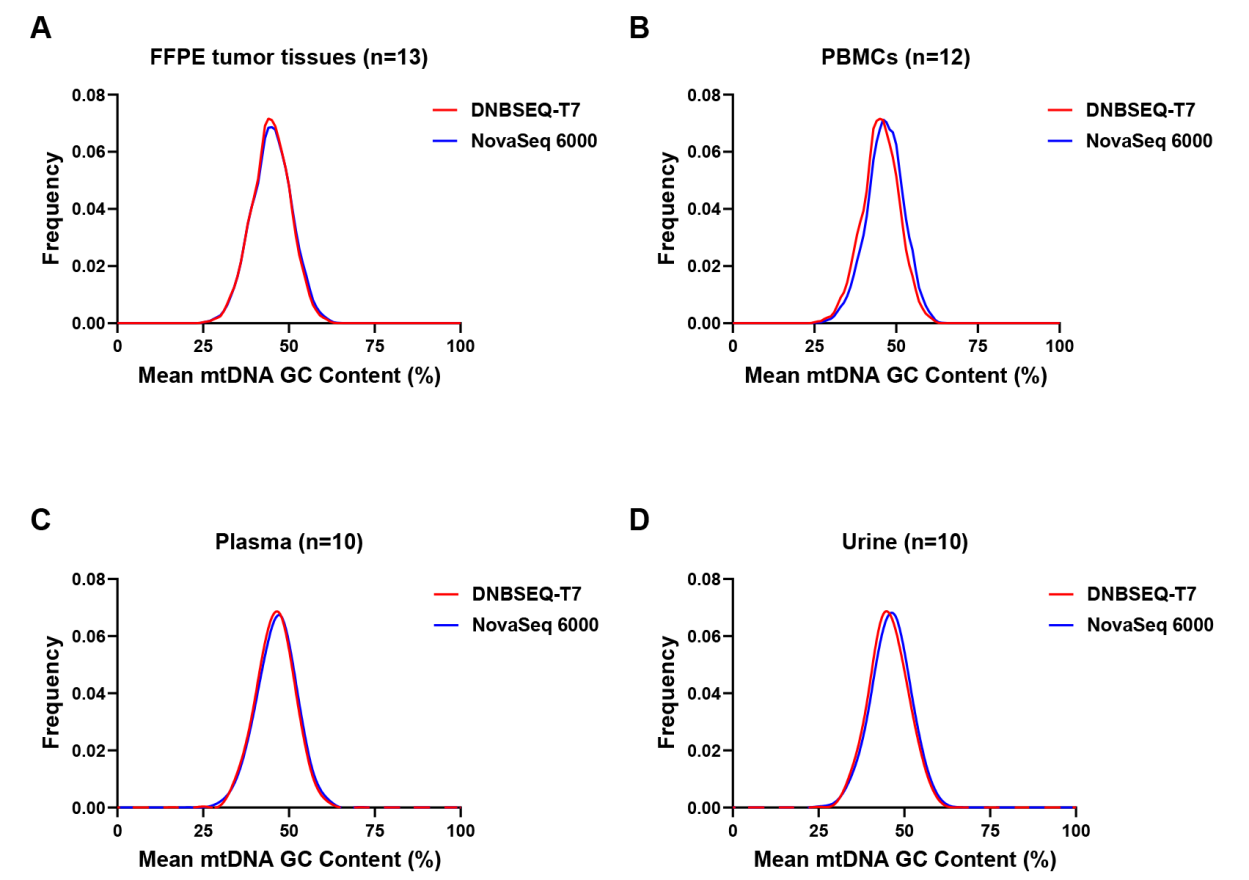
**

A-D. Comparison of the mtDNA GC content for formalin-fixed paraffin-embedded (FFPE), peripheral blood mononuclear cells (PBMCs), plasma and urine samples between the two platforms.

**Figure S3. Comparison of the depth distribution among four different sample types between the two platforms**

**
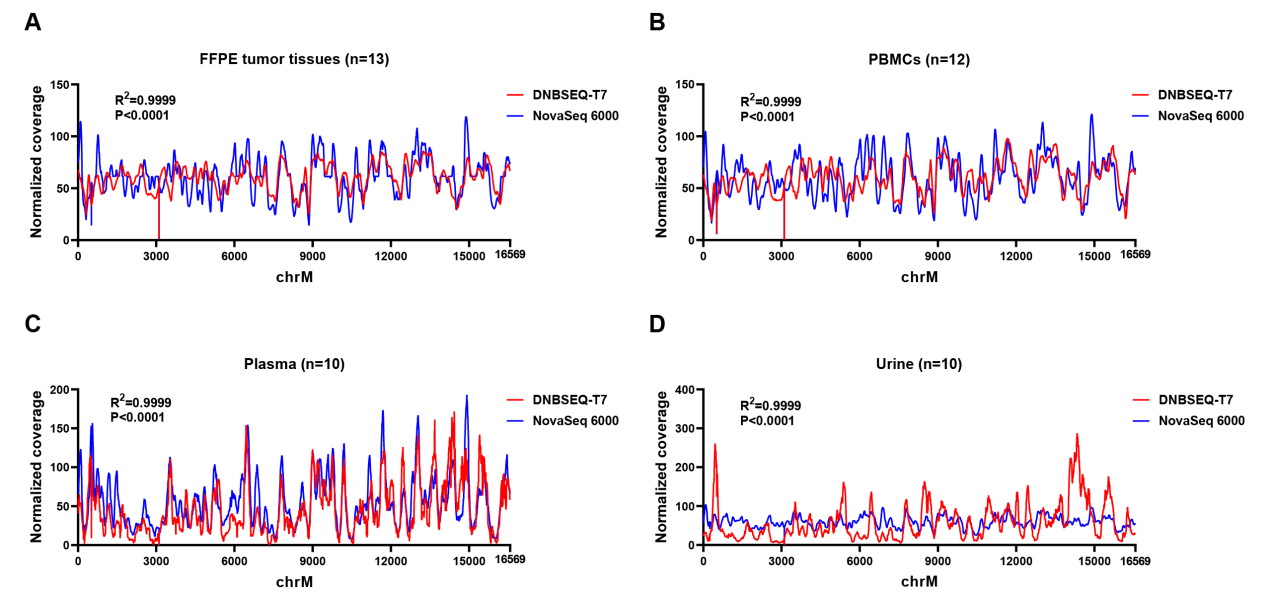
**

A-D. Comparison of the depth distribution for formalin-fixed paraffin-embedded (FFPE), peripheral blood mononuclear cells (PBMCs), plasma and urine samples between the two platforms.

**Figure S4. Comparison of the proportion of clean data among four sample types between the two platforms**

**
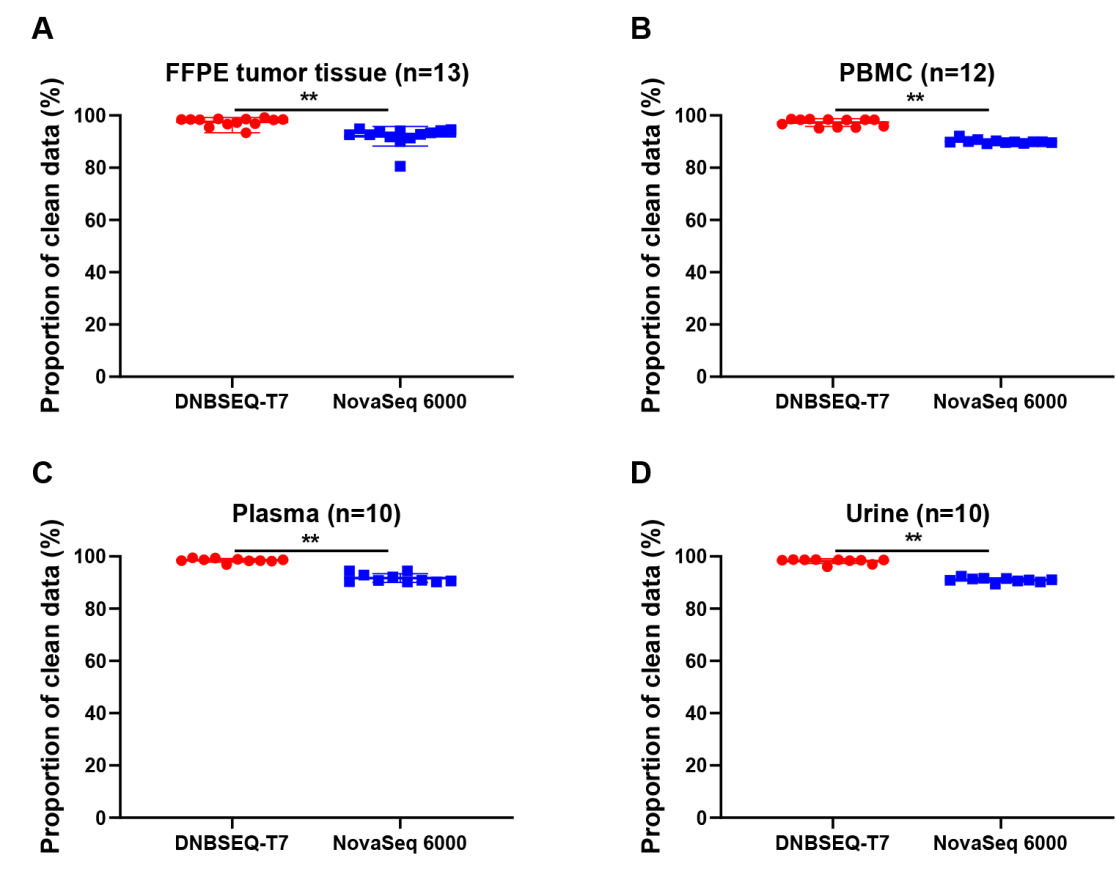
**

A-D. Comparison of the proportion of clean data for formalin-fixed paraffin-embedded (FFPE), peripheral blood mononuclear cells (PBMCs), plasma and urine samples between the two platforms.

**Figure S5. Comparison of the duplication rate among four different sample types between the two platforms**

**
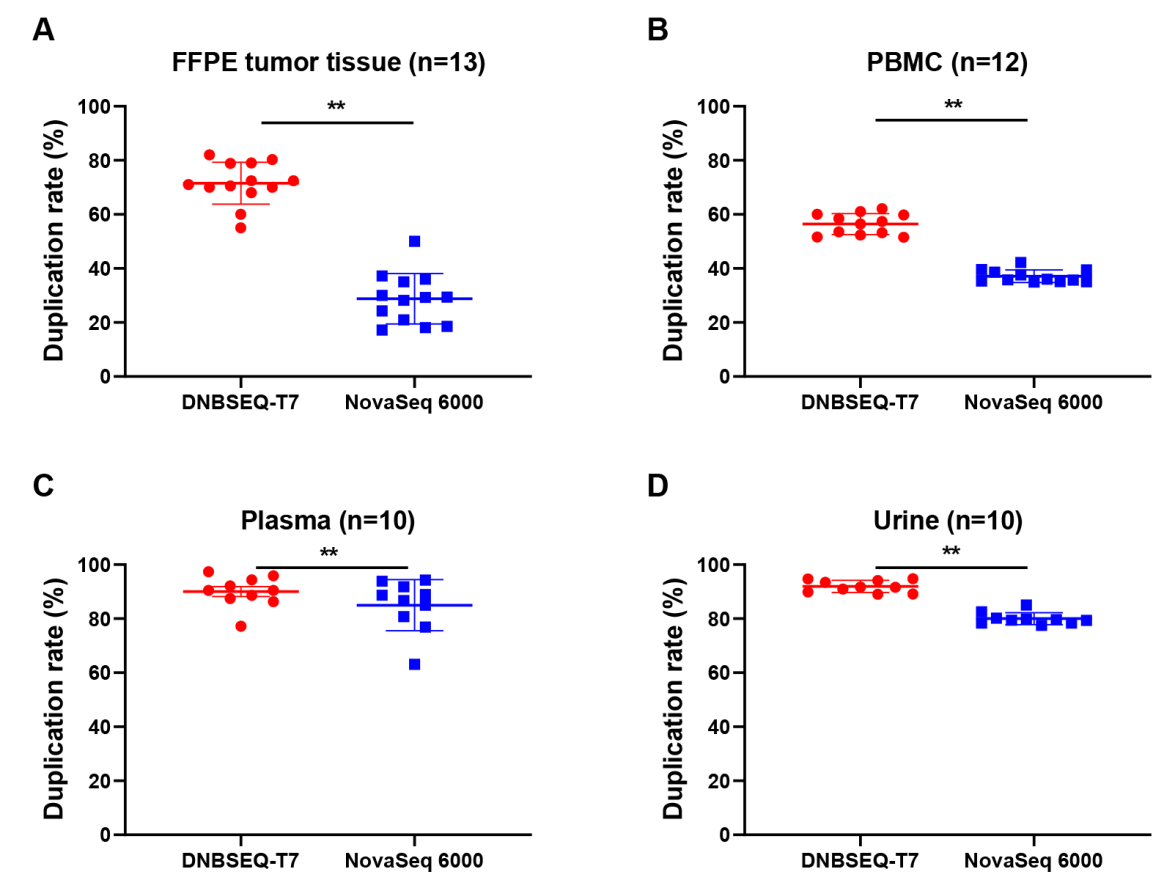
**

A-D. Comparison of the duplication rate for formalin-fixed paraffin-embedded (FFPE), peripheral blood mononuclear cells (PBMCs), plasma and urine samples between the two platforms.

**Figure S6. Comparison of the mtDNA mapping rate among four different sample types between the two platforms**

**
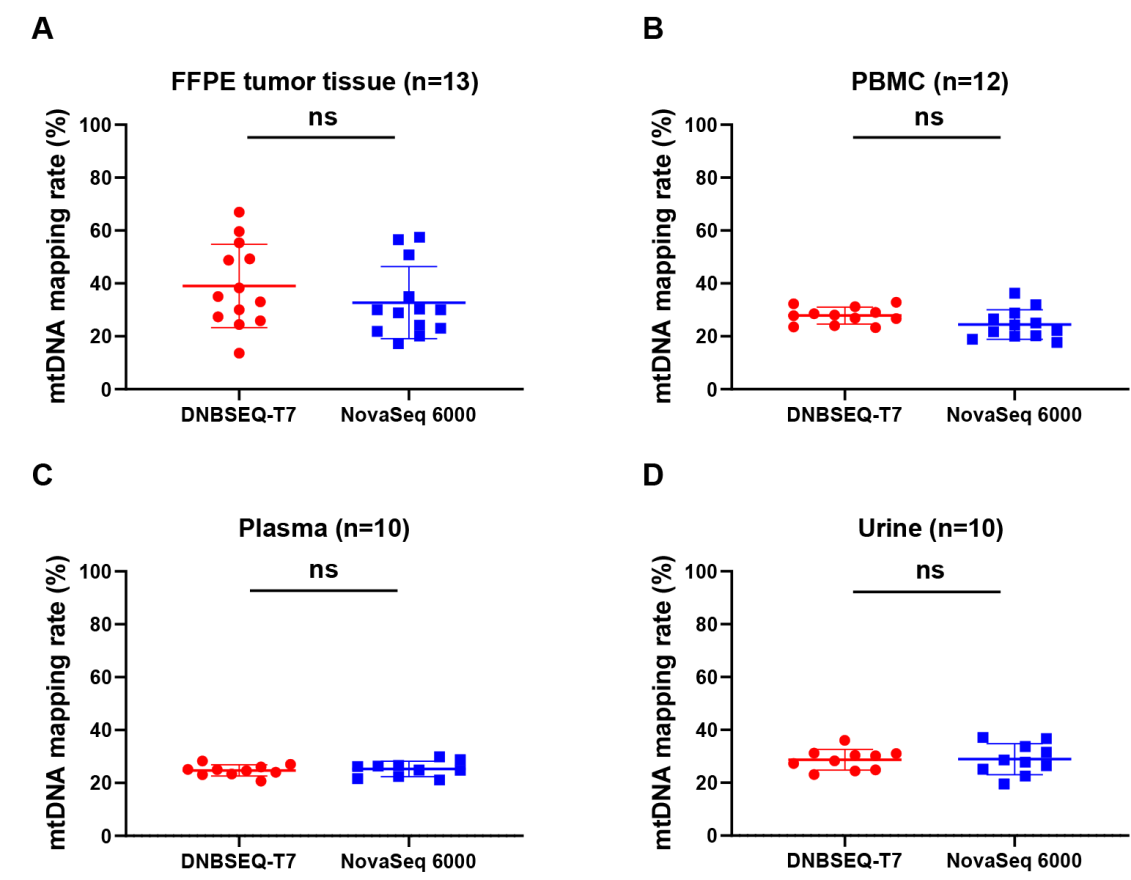
**

A-D. Comparison of the mtDNA mapping rate for formalin-fixed paraffin-embedded (FFPE), peripheral blood mononuclear cells (PBMCs), plasma and urine samples between the two platforms.

**Figure S7. Comparison of the normalized amount of sequencing data and the corresponding sequencing depth**

**
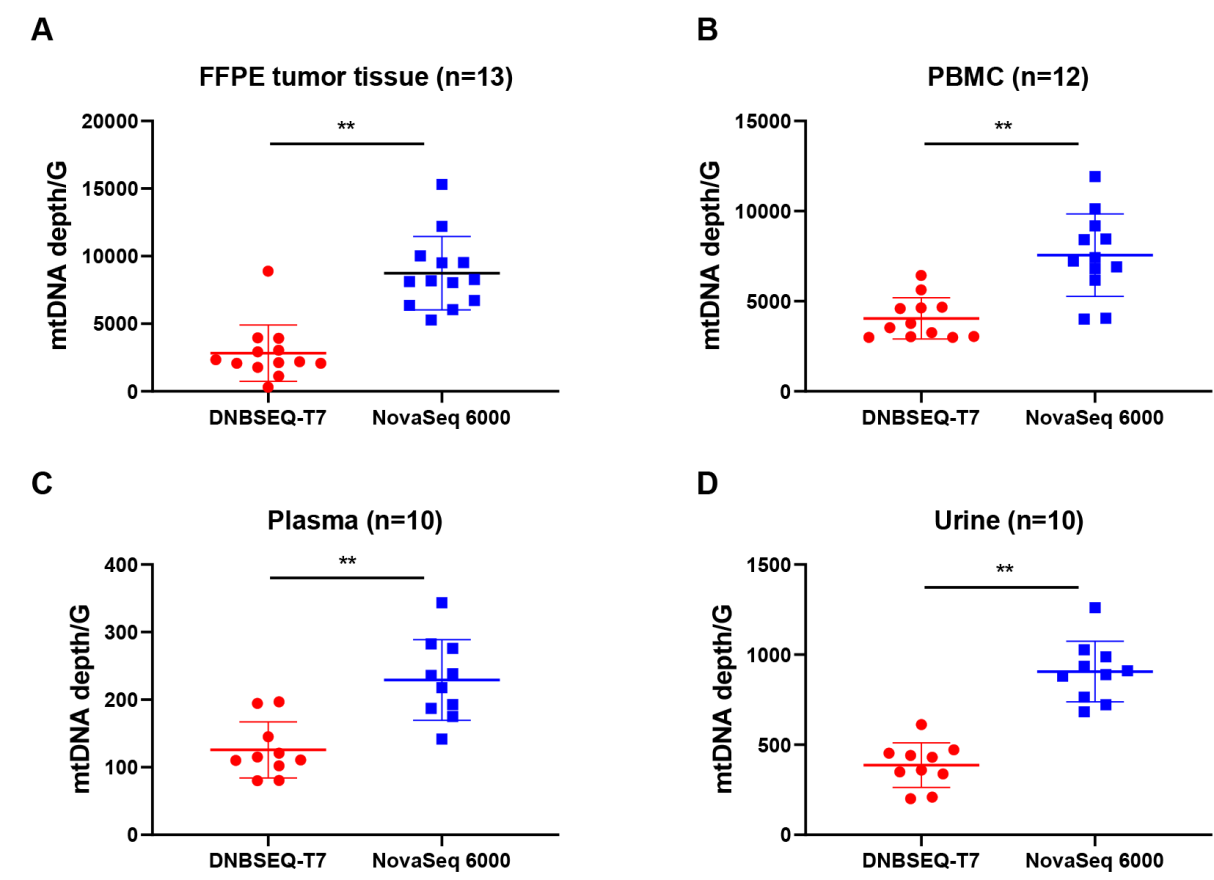
**

A-D. Comparison of the normalized amount of sequencing data and the corresponding sequencing depth for formalin-fixed paraffin-embedded (FFPE), peripheral blood mononuclear cells (PBMCs), plasma and urine samples between the two platforms.

**Figure S8. Comparison of mtDNA mutation profiles for FFPE samples**

**
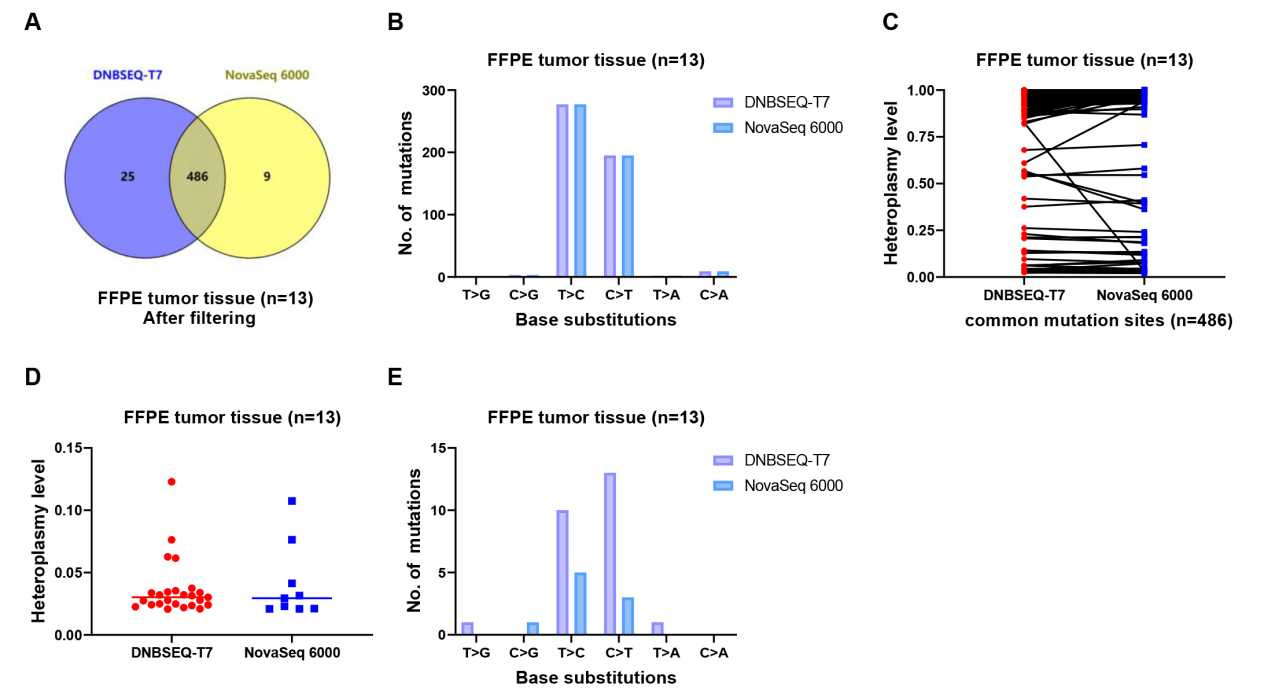
**

A-C. Comparison of the mutation numbers, the base substitution and the heteroplasmy level between the two platforms.

D-E. Comparison of the mutation density and the base substitution of platform-derived mutations

between the two platforms.

**Figure S9. Comparison of mtDNA mutation profiles for PBMC samples**

**
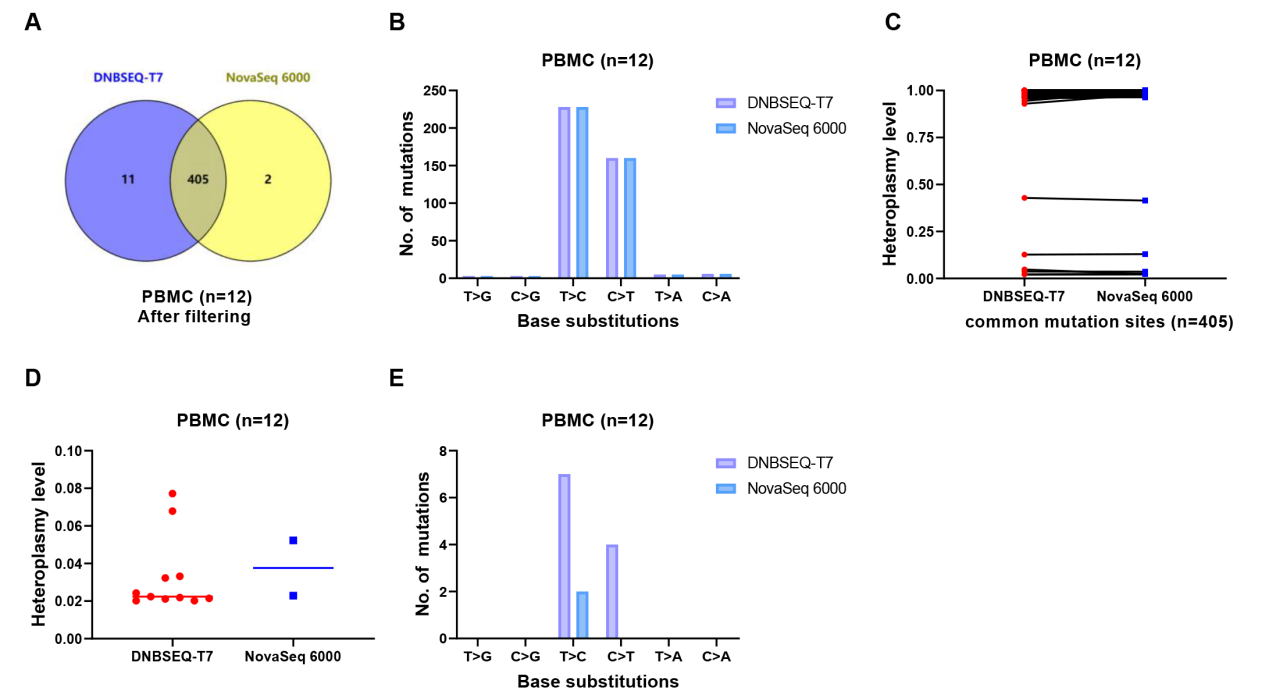
**

A-C. Comparison of the mutation numbers, the base substitution and the heteroplasmy level between the two platforms.

D-E. Comparison of the mutation density and the base substitution of platform-derived mutations

between the two platforms.

**Figure S10. Comparison of mtDNA mutation profiles for plasma samples**

**
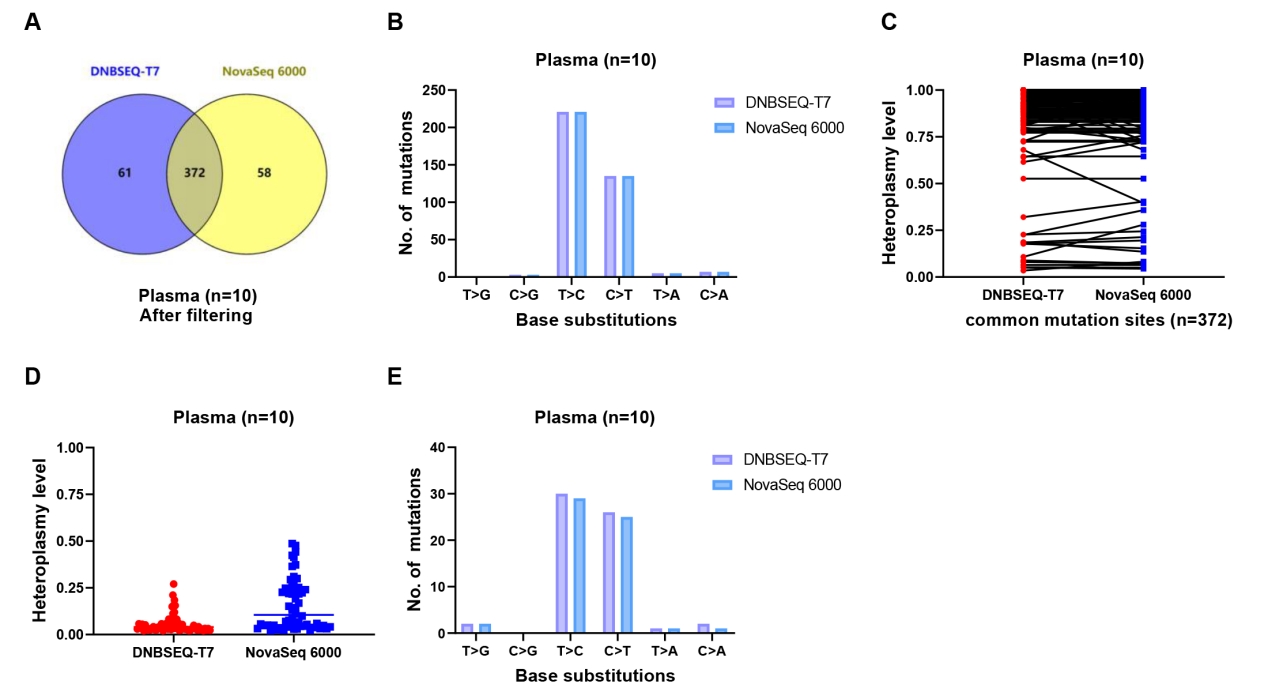
**

A-C. Comparison of the mutation numbers, the base substitution and the heteroplasmy level between the two platforms.

D-E. Comparison of the mutation density and the base substitution of platform-derived mutations

between the two platforms.

**Figure S11. Comparison of mtDNA mutation profiles for urine samples**

**
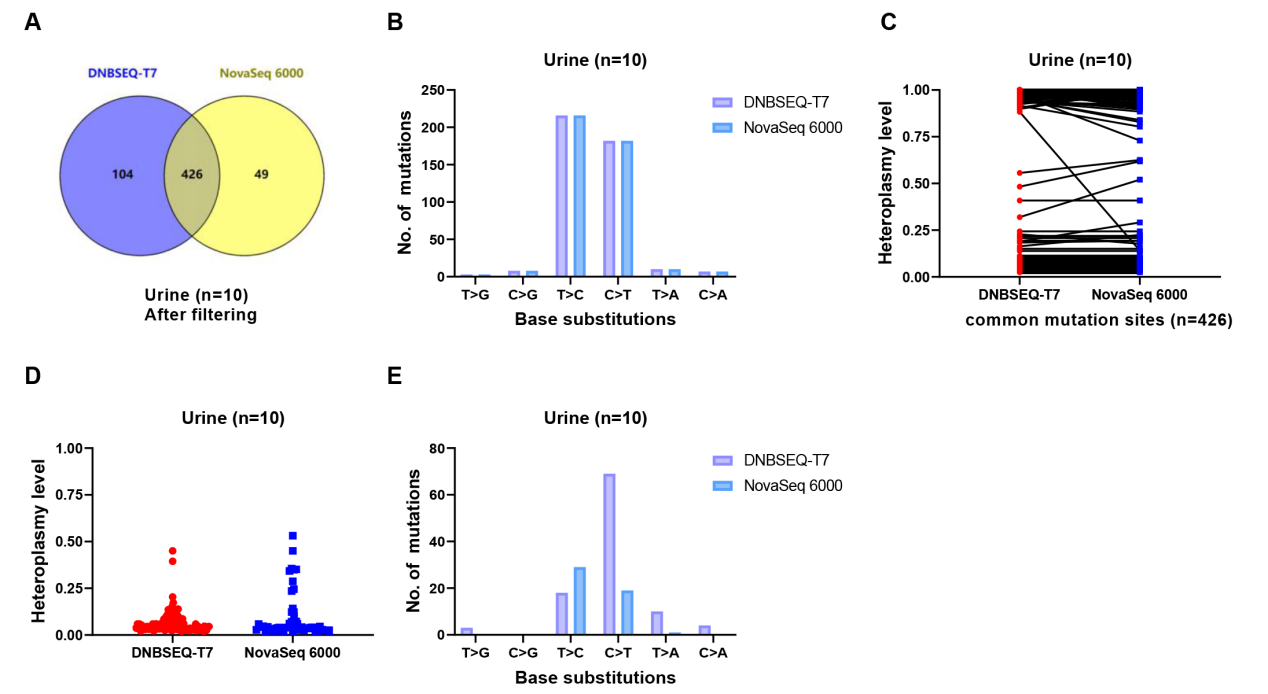
**

A-C. Comparison of the mutation numbers, the base substitution and the heteroplasmy level between the two platforms.

D-E. Comparison of the mutation density and the base substitution of platform-derived mutations

between the two platforms.
